# Efficient detection of aortic stenosis using morphological characteristics of cardiomechanical signals and heart rate variability parameters

**DOI:** 10.1038/s41598-021-03441-2

**Published:** 2021-12-10

**Authors:** Arash Shokouhmand, Nicole D. Aranoff, Elissa Driggin, Philip Green, Negar Tavassolian

**Affiliations:** 1 Department of Electrical and Computer Engineering, Stevens Institute of Technology, Hoboken, NJ 07030 USA; 2 Department of Cardiovascular Medicine, Mount Sinai Morningside Hospital, New York, NY 10025 USA; 3 The New York-Presbyterian Hospital, New York, NY 10065 USA

**Keywords:** Cardiology, Health care, Engineering

## Abstract

Recent research has shown promising results for the detection of aortic stenosis (AS) using cardio-mechanical signals. However, they are limited by two main factors: lacking physical explanations for decision-making on the existence of AS, and the need for auxiliary signals. The main goal of this paper is to address these shortcomings through a wearable inertial measurement unit (IMU), where the physical causes of AS are determined from IMU readings. To this end, we develop a framework based on seismo-cardiogram (SCG) and gyro-cardiogram (GCG) morphologies, where highly-optimized algorithms are designed to extract features deemed potentially relevant to AS. Extracted features are then analyzed through machine learning techniques for AS diagnosis. It is demonstrated that AS could be detected with 95.49–100.00% confidence. Based on the ablation study on the feature space, the GCG time-domain feature space holds higher consistency, i.e., 95.19–100.00%, with the presence of AS than HRV parameters with a low contribution of 66.00–80.00%. Furthermore, the robustness of the proposed method is evaluated by conducting analyses on the classification of the AS severity level. These analyses are resulted in a high confidence of 92.29%, demonstrating the reliability of the proposed framework. Additionally, game theory-based approaches are employed to rank the top features, among which GCG time-domain features are found to be highly consistent with both the occurrence and severity level of AS. The proposed framework contributes to reliable, low-cost wearable cardiac monitoring due to accurate performance and usage of solitary inertial sensors.

## Introduction

Aortic stenosis (AS), defined as the narrowing of the aortic valve opening, is among the most prevalent valvular heart diseases (VHDs) in developed countries^1^. The disease typically entails the left ventricle hypertrophy to compensate for blood outflow decrease due to stenosis^1,2^. Further decrease in the valve area results in the progressive overload of the left ventricular pressure, which eventually leads to severe AS unless medical treatment, i.e., transcatheter aortic valve replacement (TAVR) surgery^3,4^, is performed. Although AS is a fairly common disease, between one-third to two-thirds of the patients remain untreated as representative symptoms such as angina remain hidden at the onset of the disease^5^. Therefore, early detection of AS requires closer monitoring of cardiac activity, wherein wearable sensors could play a crucial role^6–9^.

In recent years, the performance of the cardiac system has been investigated through a variety of wearable technologies, including electrocardiography (ECG)^10^, impedance cardiography (ICG)^11,12^, photoplethysmography (PPG)^13–16^, phono-cardiography (PCG)^17,18^, ballisto-cardiography (BCG)^19,20^, seismo-cardiography (SCG)^21–23^, and gyro-cardiography (GCG)^24,25^. SCG and GCG signals, which are representatives of linear and angular vibrations of the precordium, respectively, are morphologically representative of cardio-mechanical activities^19,26,27^. They can also provide valuable information regarding cardiac timing intervals (TIs)^28–30^.

SCG/GCG signals have been applied to several applications. Several works have been dedicated to cuff-less and continuous blood pressure monitoring, where the SCG signals are used for estimating the pulse arrival time or pulse transit time^31,32^. In these cases, SCG demarcates aortic valve opening (AVO) and closure (AVC) events^32^. In addition, a deep learning approach was employed to map the SCG signal into its simultaneously-recorded BCG counterpart not measurable in a wearable setting^20^. More recently, fetal heart rate (f-HR) has been extracted using SCG and GCG modalities, where promising results were achieved in comparison with concurrently-recorded fetal cardiotocography^23^. Deploying machine learning (ML) algorithms, our research group has targeted AS detection based on the SCG/GCG technology^21,22^. In these works, the time-frequency representation of all ten second SCG/GCG segments were generated, out of which features such as the energy of frequency bands were extracted. Predictive models were then trained to classify between AS and non-AS subjects. Yet, a major drawback of the aforementioned works is the need for a reference signal, e.g., ECG^21^ or PPG^22^, for either signal segmentation or feature extraction. Moreover, there is no physical meaning behind the frequency-band energies which were used as features. A few other works have proposed SCG/GCG signal annotations without the need for a reference signal. For instance, some research works have proposed annotation methods based on time-domain analyses^33–35^, whereas some others focus on the time-frequency characteristics of SCG/GCG signals^36^.

In this paper, we propose a framework for the detection of AS by employing SCG and GCG time-domain and frequency-domain morphological features in two cases, i.e., subject-level and chunk-level analyses. In addition, the feasibility of AS detection is investigated through heart rate variability (HRV) parameters. The proposed method does not require any auxiliary reference sensors, resulting in a convenient measurement setup. Furthermore, we introduce new time-domain features to increase the confidence level of AS diagnosis. These features are extracted through a low-complexity time-domain-based approach, where no heavy computations such as wavelet transform are needed. It is shown that the proposed features are highly correlated with the occurrence of AS. In addition, the GCG time-domain features are proven to be excellent representatives of AS, which is a promising achievement for non-invasive monitoring of the cardiac system.

The rest of the paper is organized as follows. In “Methods”, we describe the motivation of the proposed methodology, the experimental protocol, the data acquisition procedure, and the ML framework. Experimental results are evaluated and compared with the literature in “Experimental results and discussion”. The paper is concluded in “Conclusions and Future Work”.

## Methods

### Motivation

**Figure 1:**
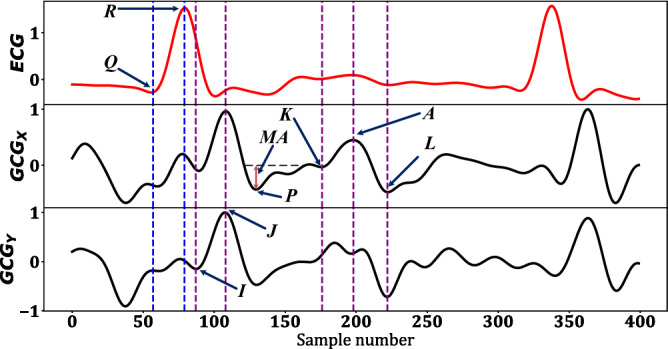
${\mathrm {GCG_{X}}}$ and ${\mathrm {GCG_{Y}}}$ fiducial points and timing intervals annotated in this work.

Following the occurrence of AS and in turn the changes in the forces against which the heart has to contract to eject blood, the morphology of the cardiac signals of AS patients are expected to differ from their normal states^2^. For instance, it has been demonstrated that the progression of stenosis is conclusively correlated with ECG ST-T wave changes in a retrospective study on 29 patients^37^. Another study has recognized prolonged Q-T wave to be an in-dependent predictor of mortality among AS patients^38^. Furthermore, the occurrence of stenosis causes HRV parameters to change accordingly^39^, which will be scrutinized as a common characteristic among AS patients in this work. Figure 1 depicts the ECG, ${\mathrm {GCG_{X}}}$, and ${\mathrm {GCG_{Y}}}$ from top to bottom, respectively, wherein ${\mathrm {GCG_{X}}}$ and ${\mathrm {GCG_{Y}}}$ are annotated according to the method discussed in the following sections. These axes of GCG provide useful information about the cardiac activity timing intervals as demonstrated in previous research works^21,22^. Hence, they are expected to provide potential insights into the diagnosis of AS.

### Experimental protocol and setup

This study includes thirty-two AS patients (sixteen males and sixteen females), and thirteen healthy subjects (seven females and six males). Among the AS patients, eleven, twelve, and nine patients were diagnosed with the severity levels of mild, moderate, and severe, respectively. The average (standard deviation) ages of the AS and healthy groups are 84.18 (9.61) years and 68.38 (17.68) years, respectively. The demographic information of the AS and non-AS groups is summarized in Table 1.

**Table 1:** Demographic information of subject groups (average ś standard deviation).

Category	Age (years)	Height (cm)	Weight (kg)
AS	84.18 (ś9.61)	164.19 (ś10.31)	72.37 (ś13.44)
Non-AS	68.38 (ś17.68)	159.31 (ś35.62)	65.94 (ś4.31)

**Figure 2:**
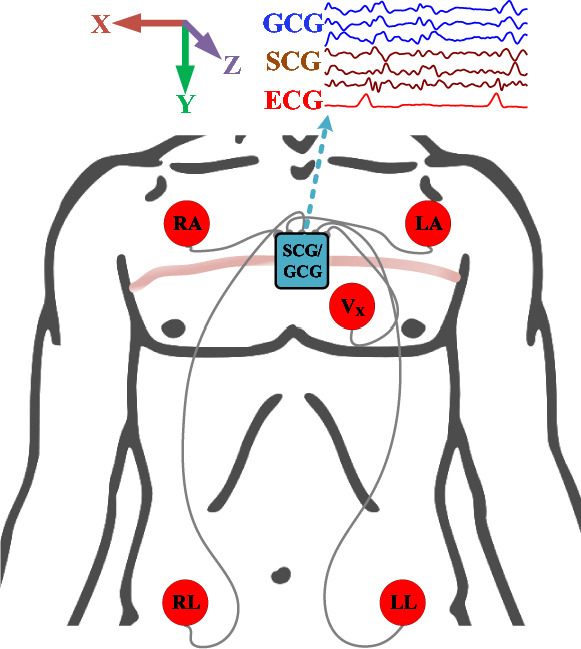
Schematic of the experimental setup for data collection.

Linear and angular vibrations of the chest wall were recorded using a commercial wearable sensor node (Shimmer3 from Shimmer sensing) secured by a band strap on the mid-sternum along the third rib. A three-axis accelerometer records SCG, and a three-axis gyroscope records GCG. Each modality is recorded in three dimensions: the x-axis corresponding to the shoulder-to-shoulder direction (along the coronal axis), the y-axis corresponding to the head-to-toe direction (along the longitudinal axis), and the z-axis corresponding to the dorso-ventral direction (along the anterior-posterior axis). In this paper, the dimensional letters X, Y, Z appended as sub-scripts to SCG/GCG denote the signal from the corresponding axis. Simultaneously, a four-lead ECG was recorded as a reference sensor. All waveforms were recorded synchronously at a 256 Hz sampling rate. The experimental setup is shown in Fig. 2.

**Figure 3:**
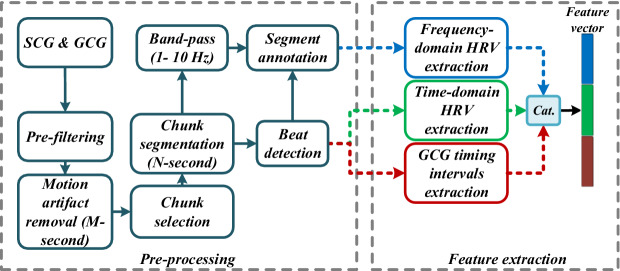
Left panel: pre-processing, right panel: feature extraction flow graphs. In the pre-processing step, motion artifacts and baseline wandering are removed from signals, which is followed by signal segmentation, peak detection, and annotation. Feature extraction is carried out for time and frequency HRV parameters as well as the GCG morphological features. Features are eventually concatenated to create a feature vector.

All data were collected at the cardiac care Unit of the Columbia University Medical Center (CUMC). The subjects were seated at rest on a bed or a chair for at least five minutes. They breathed naturally without controlling their breathing depths. The patient experimental protocol was approved by the Institutional Review Board of Columbia University Medical Center (CUMC) under protocol number AAAR4104. All methods were carried out in accordance with relevant guidelines and regulations. All participants provided written informed consent to take part in the study. Collected data were transferred to a computer and processed in a Python framework. The flow graph of the pre-processing and feature extraction procedure is illustrated in Fig. 3.

### Signal pre-processing

As shown in Fig. 3, all channels of SCG and GCG signals were initially band-pass filtered using a 4th-order Butterworth filter with cut-off frequencies of 1–45 Hz and 1–20 Hz, respectively. Subsequently, motion artifacts associated with movements during recordings were removed using a root-mean-square (RMS) filter. In most of the literature, the RMS filter is employed by applying a $M=$ 500 ms sliding window for signal segmentation, whereas in this work *M* is optimized as discussed in the following sections. Meanwhile, the segment removal threshold was selected twice the median value of the filter. It is worth noting that after motion artifact removal, signal chunks were not attached to each other, but processed separately. Therefore, if the duration of a chunk was less than *N* seconds, we would not take it into account for further processing. Here, *N* represents the size of the chunks, out of which the desired features are extracted.

**Figure 4:**
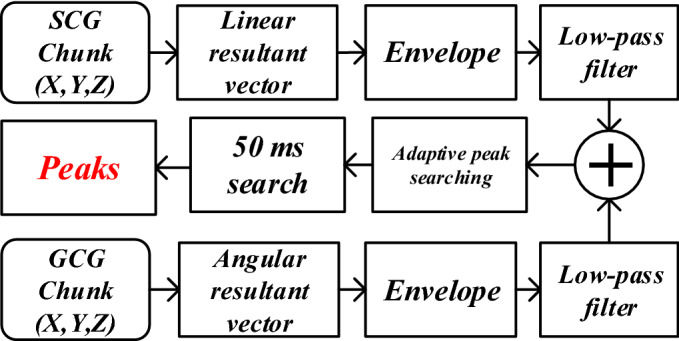
Peak detection technique based on envelope detection.

**Figure 5:**
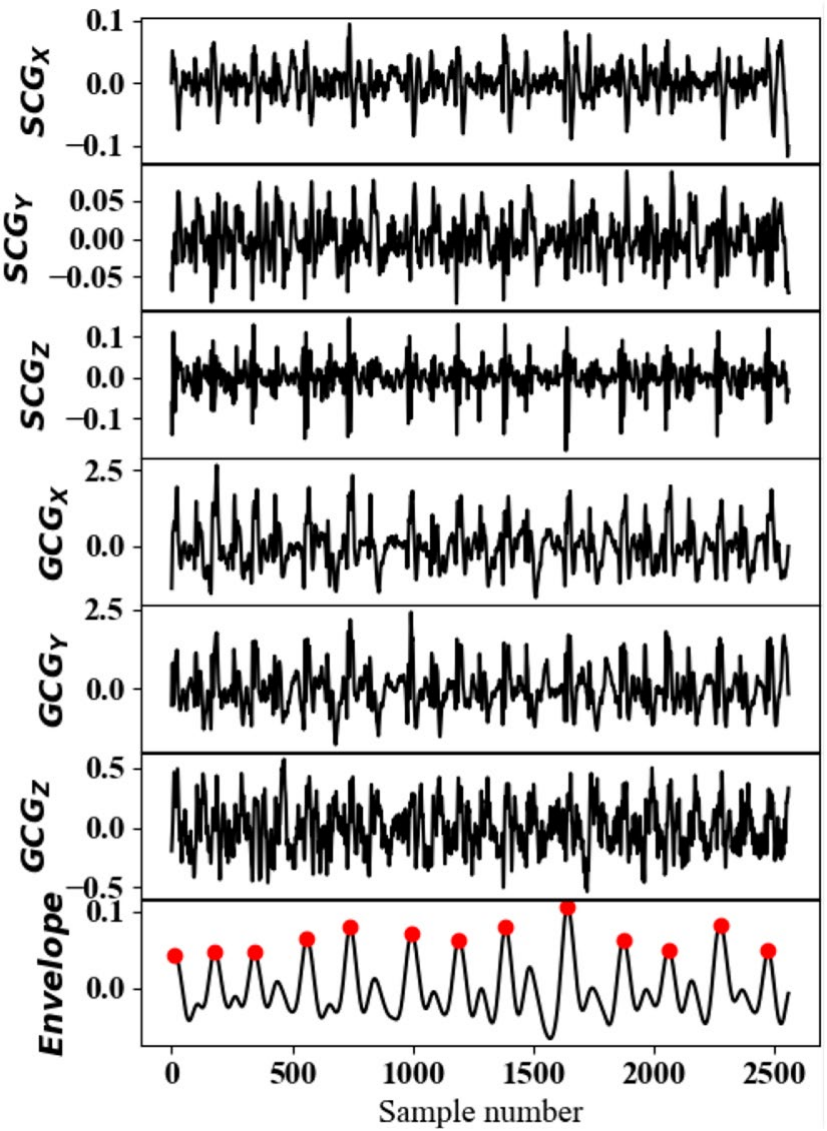
Beat detection from SCG and GCG channels, from top to bottom: ${\mathrm {SCG_{X}}}$, ${\mathrm {SCG_{Y}}}$, ${\mathrm {SCG_{Z}}}$, ${\mathrm {GCG_{X}}}$, ${\mathrm {GCG_{Y}}}$, ${\mathrm {GCG_{Z}}}$, and the envelope signal.

Inspired by^35^, a peak detection algorithm, as shown in Fig. 4, was designed to detect the ${\mathrm {GCG_{X}}}$ and ${\mathrm {GCG_{Y}}}$ peaks and annotate the fiducial points according to Fig. 1. To this end, the three axes of each of SCG and GCG were combined using the root-mean-square (RMS) function, generating the linear and angular resultant vectors, respectively, as shown in Fig. 4. It is followed then by an envelope detection technique leveraging Hilbert transform^40^. Next, a 2nd-order Butterworth low-pass filter with a cut-off frequency of 2 Hz was applied to eliminate abrupt changes in the signal. Afterwards, an adaptive peak detection algorithm based on the Pan-Tompkin method was used to discriminate the real peaks in the resulted signal from summation of linear and angular envelopes^41^. Fig. 5 illustrates the six channels of SCG and GCG modalities followed by their corresponding beats detected by the algorithm. In the end, to locate the exact positions of the peaks in each of the 6 axes, a 50-ms window, centered at the detected peaks of the summation signal, was designed to find the local maxima associated with each axis.

After peak detection within an *N*-second chunk followed by a 1-10 Hz band-pass filter, signal annotation was carried out for ${\mathrm {GCG_{X}}}$ and ${\mathrm {GCG_{Y}}}$, where I, J, K, L, and A points were characterized as proposed in^33^ and marked in Fig. 1. Along with the mentioned points, two other parameters that we named as the maximum acceleration point (P) and its corresponding amplitude, maximum acceleration (MA), were also extracted. According to the reasons mentioned in Section 2.1, we hypothesize that the time-domain features could correlate with the anatomical changes caused by the occurrence of AS.

### Feature extraction

Three types of features were extracted from SCG and GCG signals: time-domain HRV parameters, frequency-domain HRV parameters, and GCG timing interval parameters.

#### Time-domain HRV parameters

Time-domain HRV parameters provide valuable information about the cardiac activity^42^. For temporal HRV parameters, a few time-domain analyses were applied to the series of successive inter-beat intervals (IBIs). The normal-to-normal IBI (NN) is defined as the interval between consecutive J peaks in the GCG signal^43^. A few HRV parameters were extracted from the NN time series, such as the average (AVNN), the standard deviation (SDNN), root-mean-square of successive differences (RMSSD), and the proportion of the number of adjacent NN intervals whose durations differ more than 50 ms (NN50) to the total number of NNs (pNN50). It is worth mentioning that SDNN, RMSSD, and pNN50 are of great clinical significance as they allow for measuring cardiac risk, respiratory arrhythmia, and parasympathetic nervous activity^42,44^. Additionally, to further explore the impact of NN on AS detection, we introduced the median, skewness, kurtosis, entropy (ENN), self-entropy (SENN), and conditional entropy (CENN) values associated with NNs as new features. Due to the nonlinearity underlying the dynamics of HRV, we also extracted the vector angular index (VAI), the vector length index (VLI), SD1, and SD2 out of Poincare map—a scatter plot of NN at time t in terms of NN at time t + 1^45^. These features were calculated according 1 to 4:

1 $$\begin{aligned} VAI= & {} \frac{1}{B}\sum _{k=0}^{B}\left| \theta _i-45^{\circ }\right| , \end{aligned}$$

2 $$\begin{aligned} VLI= & {} \sqrt{\frac{1}{B} \sum _{k=0}^{B} \left| l_i-L\right| ^2\ }, \end{aligned}$$

3 $$\begin{aligned} SD1= & {} \mathrm {std}\left( \frac{|NN_{i+1}-NN_{i}|}{\sqrt{2}}\right) , \end{aligned}$$

4 $$\begin{aligned} SD2= & {} \mathrm {std}\left( \frac{|NN_{i+1}-NN_{i}|}{\sqrt{2}}-2\times \mathrm {AVNN}\right) , \end{aligned}$$

where $\theta _i$ denotes the angle of the $i_{th}$ scatter point with the x-axis. $l_{i}$ and *L* indicate the distance of the $i_{th}$ point to the origin and mean of distances of all *B* points to the origin, respectively.

#### Frequency-domain HRV parameters

Frequency-domain analysis was carried out based on the estimation of power spectral density (PSD) of the SCG and GCG signals, where the oscillation power of very-low-frequency (VLF), low-frequency (LF), and high-frequency (HF) bands were extracted as frequency-domain HRV parameters. It was shown that parasympathetic activities are manifested in HF (0.15–0.4 Hz), whereas sympathetic activities belong to the LF (0.04–0.15 Hz) as well as VLF (0.0033–0.04 Hz) ranges^46^. In addition to the mentioned features, the total power of PSD was calculated as an additional feature.

#### GCG timing intervals

A few timing intervals describing the cardiac system were calculated for the GCG signal. It was demonstrated that the isovolumetric contraction time (IVCT), isovolumetric relaxation time (IVRT), and left ventricular ejection time (LVET) could be estimated using J–I, L–K, and K–J^33^. Furthermore, we investigated whether MA, P, and its corresponding intervals have any impact on AS detection. Other parameters such as the intervals between each pair of the fiducial points depicted in Fig. 1 along with their mean, median, standard deviation, skewness, and kurtosis values were extracted as auxiliary features. The logic behind such an exhaustive feature extraction is to characterize the most relevant GCG timing intervals resulting in the highest accuracy of AS diagnosis.

The extracted features are summarized in Table 2.

**Table 2:** Feature space by group types.

Time HRV features	GCG features*	Frequency HRV features
AVNN	IVCT (J–I)	VLF
SDNN	LVET (K–J)	LF
RMSSD	IVRT (L–K)	HF
pNN50	L–I	LF/HF
SD1	L–J	Total power
SD2	K–I	
SD1/SD2	P–I	
VAI	P–J	
VLI	K–-P	
ENN	L–P	
CENN	MA	
SENN		
NN skewness		
NN kurtosis		
NN median		

*Mean, median, standard deviation, skewness, kurtosis, entropy, min, and max were calculated for every parameter in this column.

### Training and hyperparameter optimization

**Figure 6:**
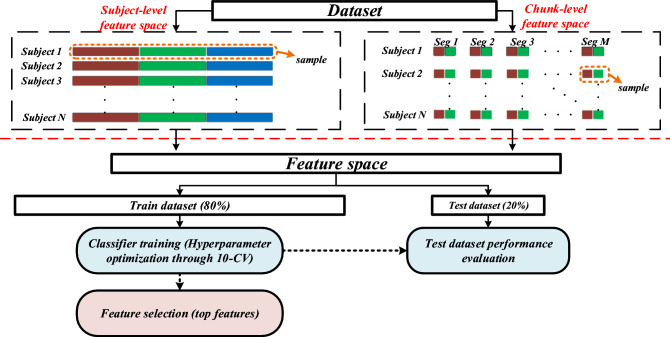
Subject-level and chunk-level datasets. In the subject-level dataset, each subject is considered a sample. In the chunk-level dataset, every chunk represents a sample. The hyperparameters of ML classifiers are fine-tuned through a 10-fold cross-validation. The trained models are evaluated using the remaining 20% of datasets. The top features are ranked according to their contribution to the classifier output.

Figure 6 shows the general schematic of the proposed machine learning framework including feature engineering, data split, training, and feature selection. As demonstrated in this figure, AS detection was performed in two different cases: subject-level and chunk-level. The former implies using each subject as a single sample, whereas the latter suggests each signal chunk as a sample for training the predictive models. In the chunk-level feature space, frequency-domain features were avoided since the chunk size is not long enough to accurately calculate the spectral parameters, while the whole signal could be used to measure the spectral features. Regardless of the scenario, the entire feature space was split into two parts, training (80%) and test (20%) datasets. Following the data split, we trained the predictive models, where the hyperparameters were optimized through leave-one-out 10-fold cross-validation (10-CV). For subject-level 10-CV, 0.1 of the subjects were held out at each fold, and the model continued to be optimized using the rest 0.9 held-in subjects. However, in the chunk-level 10-CV training, 0.1 of the total chunks were held out. It should be noted that both training and hyperparameter optimization were carried out using only the training part in each case.

### Classification techniques and evaluation metrics

Given the two datasets, we used four machine learning techniques for the diagnosis of AS: decision tree (DT), random forest (RF), extreme gradient boosting (XGBoost), and support vector machine (SVM)^47,48^. These classifiers are widely employed in a variety of biomedical applications including opioid patients monitoring^49^, heart failure prediction^50^, and cancer prognosis^51^ due to their robustness.

During the 10-CV, the hyperparameters of each model were tuned in terms of performance. Table 3 presents the parameters with respect to which the ML models were optimized. For instance, the DT model was optimized in terms of the maximum depth of the tree, the minimum number of samples in a leaf, the minimum number of samples for splitting a node, the maximum number of features, and the criterion used for root selection. Similar parameters were optimized for RF and XGBoost. For the case of XGBoost, however, the model should also be tuned in terms of the learning rate, since training the XGBoost follows a gradient-based pattern. Furthermore, all predictive models were optimized in terms of class weights to tackle the data imbalance.

**Table 3:** Hyperparameters of the ML models.

DT	RF	XGBoost	SVM
Maximum depth	Maximum depth	Maximum depth	Regularization parameter
Minimum samples per leaf	Minimum samples per leaf	Minimum samples per leaf	Kernel
Minimum split samples	Minimum split samples	Minimum split samples	Kernel coefficient
Maximum features	Maximum features	Maximum features	Class weights
Criterion	Criterion	Criterion	
Class weights	Number of estimators	Learning rate	
	Class weights	Class weights	

The following metrics were calculated to evaluate the performance of the classification algorithms: precision (PR), recall (RE), accuracy (AC), and F1-score (F1). AC is a simple metric that measures the accuracy of the model in prediction, and deals only with true predictions. However, it does not provide information for the cases where the model misclassifies a sample. PR and RE serve to deal with this problem by introducing false alarms and missed rates, respectively. As a combination of PR and RE, F1 offers a more comprehensive understanding of the performance, which was used for filter optimization as well.

### Filter optimization

**Figure 7:**
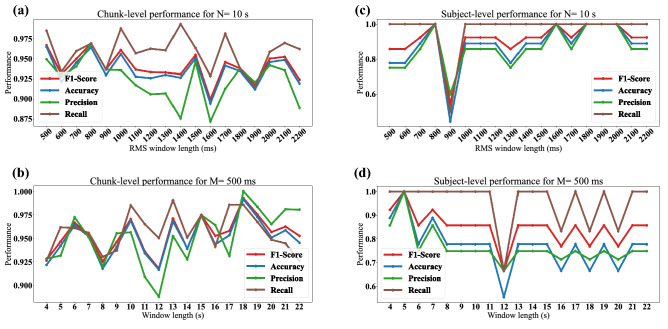
Performance evaluation of XGBoost for filters based on literature. (**a**) Chunk-level performance for $N=$ 10 s, (**b**) chunk-level performance for $M=$ 500 ms, (**c**) subject-level performance for $N=$ 10 s, and (**d**) subject-level performance for $M=$ 500 ms.

As explained in previous sections, we employed an RMS filter for motion artifact removal with length *M* (ms). It was also mentioned that we segmented the GCG signals into *N*-second windows. These parameters tend to be fixed to $M=$ 500 ms and $N=$ 10 s in the literature^22,52^, which are experimental numbers. We analyzed the effects of changing these parameters on the performance of XGBoost model. To this end, the performance metrics were assessed in terms of different values for *M* and *N* in Fig. 7, separately. Fig. 7a and b illustrate the performance for $N=$ 10 s in terms of different values for *M* and $M=$ 500 ms in terms of different values for *N* at the chunk-level analyses, respectively. The same characteristics are investigated in Fig. 7c and d for the subject-level. As depicted in these figures, suggesting optimum values for *M* and *N* is not a straightforward process. For instance, Fig. 7a suggests $N=$ 10 s and $M=$ 500 ms for the maximum F1-score, whereas in Fig. 7b in order to achieve the best performance, *N* should hold 18 s, which in turn implies that these parameters need to be tuned at the same time. We, therefore, defined an optimization problem to simultaneously optimize the values of *M* and *N*. The optimization problem is defined as follows:

5 $$\begin{aligned} \begin{matrix} \displaystyle {M^*,N^*} = {arg\,max}_{M,N} F_{1}(M,N) \\ \text {s.t.}\ &{}M \in [100 , 2000 ]\ \mathrm {ms},\ N \in [2, 25]\ \mathrm {s}, \\ &{} \displaystyle \\ \end{matrix} \end{aligned}$$

where $M^*$ and $N^*$ indicate the optimum values resulting in the highest possible F1-score. To solve the optimization problem, the Bayesian optimization method is employed. This optimization method introduces a surrogate for the cost function and measures its uncertainty by a Bayesian learning technique and a Gaussian process regression. It then defines an acquisition function from the surrogate to determine the sampling locations and find the optimum values^53^. In this way, an end-to-end training procedure results in the optimized objective function and its corresponding filter parameters.

## Experimental results and discussion

In this section, the experimental results are discussed and compared with the literature.

### Datasets, features, and filter optimization

As mentioned earlier, two datasets were provided. The subject-level dataset included 45 subjects, where 36 of them were used to train the models, and the remaining 9 were held out for the test datasets. The held-out group consists of two mild AS, one moderate AS, two severe AS, and four healthy individuals. Out of 36 training subjects, at each fold of 10-CV, 32 subjects were held in for training and 4 subjects were held out for hyperparameter optimization. Per each subject, mean, median, standard deviation, skewness, kurtosis, entropy, min, and max (8 features) were calculated for every 11 GCG features tabulated in Table 2. Thus, the total number of GCG features was 11Œ8 = 88. Furthermore, 5 frequency-domain HRV parameters were extracted for each channel axis (6 axes) per subject, amounting to 6Œ5=30 features. Besides, 15 time-domain HRV parameters were extracted out of NNs for each subject. Therefore, the total number of extracted features for each sample in the subject-level feature space was 123. Chunk-level feature space included 1272 chunks, out of which 88 GCG timing parameters as well as 15 time-domain HRVs (total of 103 features) were calculated. For training the chunk-level dataset, 255 and 1017 chunks were used for test and training, respectively. Subsequently, 102 chunks were held out for 10-CV hyperparameter optimization, whereas 915 chunks were held in for fine-tuning the model.

The RMS filter length and chunk window length were optimized by the Bayesian optimization technique through the process of training. It should be noted that these parameters were optimized by involving the training dataset, but not the test dataset. The optimum values achieved for filter parameters were $M^*$=1582 ms and $N^*$=11.2 s. The RMS filter length is three times the value proposed in the literature, i.e., $M=$500 ms, whereas the length of the chunk is almost consistent with the literature.

### Feature selection for models

We conducted both the Shapley Additive exPlanations (SHAP) technique, a game theory-based approach for interpreting the output of a model in terms of the input feature space, and the feature-importance f-score from XGBoost to provide an explainable model^54^. SHAP is employed as it calculates the feature importance in terms of the impact of every single observation on the output performance, whereas the f-score from XGBoost represents the importance of a feature for the whole dataset. The score for each feature in SHAP method is calculated by the following formula:

6 $$\begin{aligned} \alpha _i = \sum _{\gamma \ \subset \ \Gamma \ \setminus \ i} \frac{|\gamma |!(|\Gamma | - |\gamma | - 1)!}{|\Gamma |!}[F1(\gamma \cup i)-F1(\gamma )], \end{aligned}$$

where $\alpha _i$, $\gamma$, i, and $\Gamma$ denote the score value of the $i_{th}$ feature, any subset of feature space excluding the $i_{th}$ feature, $i_{th}$ feature, and the entire feature space, respectively. *F*1 also represents the F1-score, where $[F1(\gamma \cup i)-F1(\gamma )]$ indicates the difference of F1-score resulting from incorporating the $i_{th}$ feature within the feature space. For every feature, $\alpha _i$ is calculated for all samples in the training dataset and the resulting values are averaged to obtain the feature score. F-score by XGBoost, on the other hand, evaluates each feature based on its impact on the output for all samples at once. Thus, SHAP values validate the feature ranking obtained by XGBoost feature importance and vice versa. That is, if the scores from both cases demonstrated an agreement, one can conclude the model is robust enough for the prediction task. In the following sections, we assess the importance of the features through both XGBoost and SHAP values.

### Performance evaluation

After training and hyperparameter optimization, the performance of the proposed method is evaluated by applying the model on the test dataset to predict the existence of AS. Next, by comparing the predicted values and the true labels, the performance of the method is reported using metrics introduced in the previous section. Table 4 summarizes the performance results for the two datasets. The optimized method was reported by 100% accuracy, 100% F1-score, 100.00% precision, and 100.00% recall for DT, XGBoost, and SVM at the subject-level. As reported, DT outperforms RF, indicating 77.78% accuracy and 87.50% F1-score for the models with literature-based parameters ($M=$ 500 ms and $N=$ 10 s). Similarly, DT suggests a higher F1-score in comparison to RF, i.e., 100.00% vs. 80.00%, for the models with optimized parameters ($M^*$= 1582 ms and $N^*$= 11.2 s). Having a closer look at the performance of the models with optimized parameters and literature-based ones at subject-level analyses, the accuracy and F1-score of DT were improved, respectively, by 22.22% and 12.50%. Furthermore, XGBoost shows 85.71% F1-score, which is the highest performance among the models with literature-based parameters at the subject level. Yet, XGBoost with the optimized filter parameters implies 100.00% of accuracy and F1-score at the subject level. Such an improvement demonstrates the functionality of filter optimization. According to the optimized chunk-level analyses in Table 4, XGBoost and RF outperform other methods, suggesting 96.49% and 90.34% of F1-score, respectively. These values offer a more robust performance than the non-optimized F1-score, i.e., 95.49% for XGBoost and 86.87% for RF. Furthermore, the F1-score of the optimized XGBoost model degrades from 96.49% to 95.49% when it uses the literature parameters, denoting an increase in FP as confirmed by the decrease in recall (97.35% vs. 95.17%). In the chunk-level dataset, XGBoost surpassed the other methods at high margins, i.e., 7.64%, 5.14%, 4.391%, and 6.15% for accuracy, precision, recall, and F1-score, respectively. RF performs better than DT in terms of F1-score (90.34% vs. 88.89%), suggesting that the larger the dataset, the easier the generalizability of the RF model to the test datasets. Nevertheless, XGBoost offers a superior predictive characteristic than other methods at the chunk level. Additionally, the other optimized models at chunk-level analyses outperform their non-optimized versions in terms of F1-score by an acceptable margin (DT: 88.89% vs. 84.14%, RF: 90.34% vs. 86.87%, and SVM: 90.09% vs. 86.81%).

**Table 4:** Performance evaluation of the proposed method for the optimized and non-optimized models.

Classification level	Subject (optimized parameters)				Chunk (optimized parameters)			
Classifier	Accuracy	Precision	Recall	F1-score	Accuracy	Precision	Recall	F1-score
	(%)	(%)	(%)	(%)	(%)	(%)	(%)	(%)
DT	100.00	100.00	100.00	100.00	87.94	90.51	87.32	88.89
RF	66.66	66.66	100.00	80.00	88.21	87.88	92.95	90.34
XGBoost	**100.00**	**100.00**	**100.00**	**100.00**	**96.36**	**95.65**	**97.35**	**96.49**
SVM	100.00	100.00	100.00	100.00	88.72	87.42	92.96	90.09

Classification level	Subject (Literature-based parameters)				Chunk (Literature-based parameters)			
Classifier	Accuracy	Precision	Recall	F1-score	Accuracy	Precision	Recall	F1-score
	(%)	(%)	(%)	(%)	(%)	(%)	(%)	(%)
DT	77.78	87.50	87.50	87.50	81.96	84.14	84.14	84.14
RF	66.66	66.66	100.00	80.00	84.71	84.87	88.97	86.87
XGBoost	**77.78**	**75.00**	**100.00**	**85.71**	**94.89**	**95.83**	**95.17**	**95.49**
SVM	77.78	71.43	100.00	83.33	85.09	87.41	86.21	86.81

Significant values are in bold.

**Figure 8:**
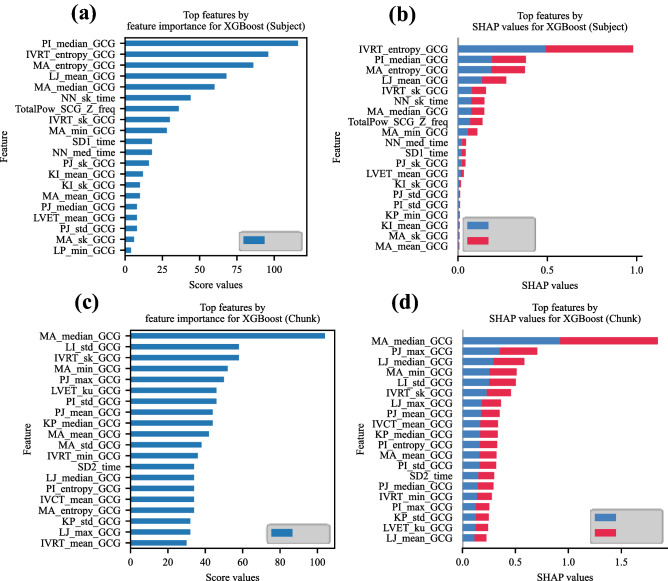
Top features by feature importance of XGBoost and SHAP. (**a**) Feature importance for subject level, (**b**) SHAP values for subject level, (**c**) feature importance for chunk level, and (**d**) SHAP values for chunk level. 90% and 85% consistencies are found between feature importance and SHAP values at subject-level and chunk-level, respectively.

Figure 8 depicts the top 20 features for both chunk and subject levels. Figure 8a and b illustrate the top 20 features achieved by XGBoost f-score and SHAP values for subject-level analyses, respectively. Figure 8c and d show the same order for chunk-level analyses. Among the top 20 features of subject-level analyses, eighteen features (90.00%) are consistent between the two criteria of importance, whereas seventeen features (85.00%) are reported as the common ones between the two methods at the subject level. According to Fig. 8b, among the top features, sixteen belong to GCG morphological features, three to time-domain HRV, and one to frequency-domain HRV parameters, demonstrating the higher representativeness of GCG features in comparison to others. Moreover, the highest occurrences belong to MA and P-point-included intervals, each with five features among the top-20’s. From the time-domain HRV parameters, NN shows the highest correlation with AS, whereas the only feature is the total power of ${\mathrm {SCG_{Z}}}$ from the frequency-domain HRV space. For the chunk-level analyses, nineteen features belong to the GCG time-domain features, whereas only one feature, i.e., NN, belongs to the time-domain HRV feature space. Among the top GCG features, MA, P-J, and L-J repeat three times, suggesting the highest frequencies among the top 20 features. Furthermore, the top features presented in Fig. 8b and d almost equally contribute to making a decision on AS and healthy states (blue and red bars), denoting that the top features are representative of both classes.

### Ablation study on feature space

An ablation study was conducted on feature spaces to investigate the predictive performance of AS diagnosis. XGBoost was used in this section due to its superior performance on both datasets. The optimized parameters were used for both subject-level and chunk-level classification tasks. Three feature spaces for the subject-level and two feature spaces for the chunk-level (frequency-domain HRV parameters are not included for chunk-level) datasets were assessed. Thus, the performance of the model was evaluated for GCG time-domain parameters, time-domain HRV, and frequency-domain HRV parameters separately. The results are summarized in Table 5. As shown for the chunk-level study, the best performance belongs to GCG timing intervals (95.19% F1-score), whereas the weakest performance is achieved by time-domain HRV parameters (74.56% F1-score). Furthermore, frequency-domain HRV introduces more relevant information for AS detection compared to time-domain HRV features at the subject-level (80.00% vs. 60.00%). This implies that frequency-domain HRV parameters carry more information regarding AS than the time-domain HRV parameters (accuracy: 66.67% vs. 55.56%).

**Table 5:** Feature ablation evaluation using XGBoost.

Classification level	Subject				Chunk			
Feature type	Accuracy	Precision	Recall	F1-score	Accuracy	Precision	Recall	F1-score
	(%)	(%)	(%)	(%)	(%)	(%)	(%)	(%)
GCG intervals	**100.00**	**100.00**	**100.00**	**100.00**	**95.00**	**93.97**	**96.46**	**95.19**
Time HRV	55.56	75.00	50.00	60.00	73.64	73.91	75.22	74.56
Frequency HRV	66.67	66.67	100.00	80.00	N/A	N/A	N/A	N/A

Significant values are in bold.

Furthermore, the top five features for the datasets in the ablation study are extracted and shown in Table 6. According to the time-domain HRV parameters listed for chunk-level and subject-level studies, NN, SD1, and SD1/SD2 contribute more than others to AS diagnosis, whereas the top features from frequency-domain HRV features include the total power, LF/HF ratio, and HF power.Interestingly, three GCG features out of five in both cases are associated with MA and the intervals involving the P-point. Also, IVRT is ranked twice as the best feature for the subject level, showing the importance of this feature.

**Table 6:** Top-five features of ablation study by SHAP values.

Subject-level			Chunk-level		
Time HRV features	GCG features	Frequency HRV	Time HRV features	GCG features	Frequency HRV
NN_sk_time	IVRT_entropy_GCG	TotalPow_GCG_X_freq	NN_med_time	MA_median_GCG	N/A
NN_ku_time	IVRT_sk_GCG	lfhfRatio_GCG_Z_freq	AVNN_time	LJ_median_GCG	N/A
SD2_time	PI_median_GCG	hf_HRV_GCG_Y_freq	SDNN_time	PJ_max_GCG	N/A
SD1/SD2_time	MA_min_GCG	TotalPow_GCG_Z_freq	SD1/SD2_time	MA_mean_GCG	N/A
AVNN_time	MA_entropy_GCG	hf_HRV_GCG_X_freq	SD1_time	LJ_max_GCG	N/A

### Classification of severity level based on per-patient basis

In this section, the robustness of the proposed method is evaluated for diagnosing the severity level of aortic stenosis on a per-patient basis. For this purpose, a 4-class classification was conducted to observe the failure modes of the proposed method. The classification involves the healthy group as well as mild, moderate, and severe aortic stenosis cases. For a 4-class classification, we augmented the size of the dataset by considering 50% of overlap between every two consecutive signal chunks. This practice helps the predictive model to better be generalized to the test dataset. As a result, a total of 2336 chunks were generated, of which 1868 and 468 were used for training and test datasets, respectively.

**Figure 9:**
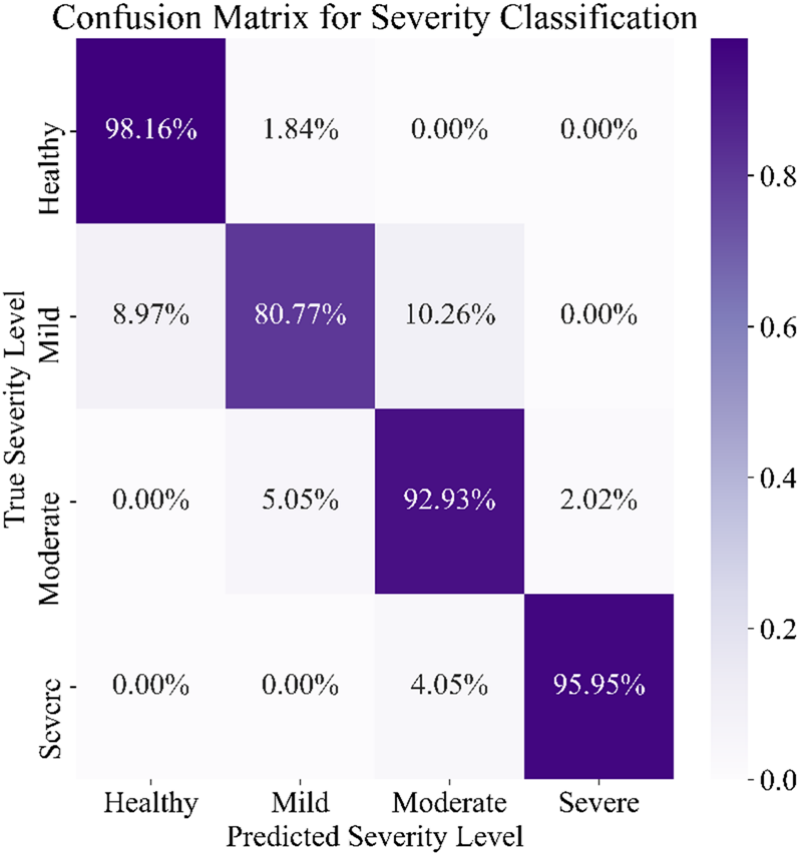
The Confusion matrix for a 4-class classification.

The 4-class classification results suggest 92.72% precision, 91.95% recall, 93.80% accuracy, and 92.29% F1-score. As for the F1-score, the performance of the 4-class classification has been slightly degraded compared to the binary classification for aortic stenosis (92.29% vs. 96.49%). This small difference has occurred due to the small size of the dataset with respect to the number of classes (4 classes). To investigate the failure modes, the classification results are summarized in a confusion matrix depicted in Fig. 9. As illustrated in this figure, 98.16% of the healthy group were classified correctly, whereas the remaining 1.84% were classified as mild cases. According to the confusion matrix, the mild, moderate, and severe cases were classified correctly with the rates of 80.77%, 92.93%, and 95.95%, respectively. These results suggest that severe patients’ recordings hold distinctive characteristics which serve to distinguish them from other cases. The 4.05% misclassified severe cases were reported as moderate cases. This misclassification has happened due to the morphological similarities between moderate and severe cases. Interestingly, a severe case was never misclassified as a healthy case or mild AS, which demonstrates the robustness of the proposed feature space with respect to the number of classes. As for the mild severity, 8.97% and 10.26% were classified as healthy and moderate cases, respectively, which could have been caused by either the size of the training dataset, or the similar morphological characteristics of mild AS with healthy and moderate cases. As for the moderate cases, a small aggregate of 7.07% was misclassified as either mild or severe, which proves the practicality of the proposed framework. Having considered the aforementioned points, it is concluded that the proposed method offers high reliability in detecting and classifying AS.

**Figure 10:**
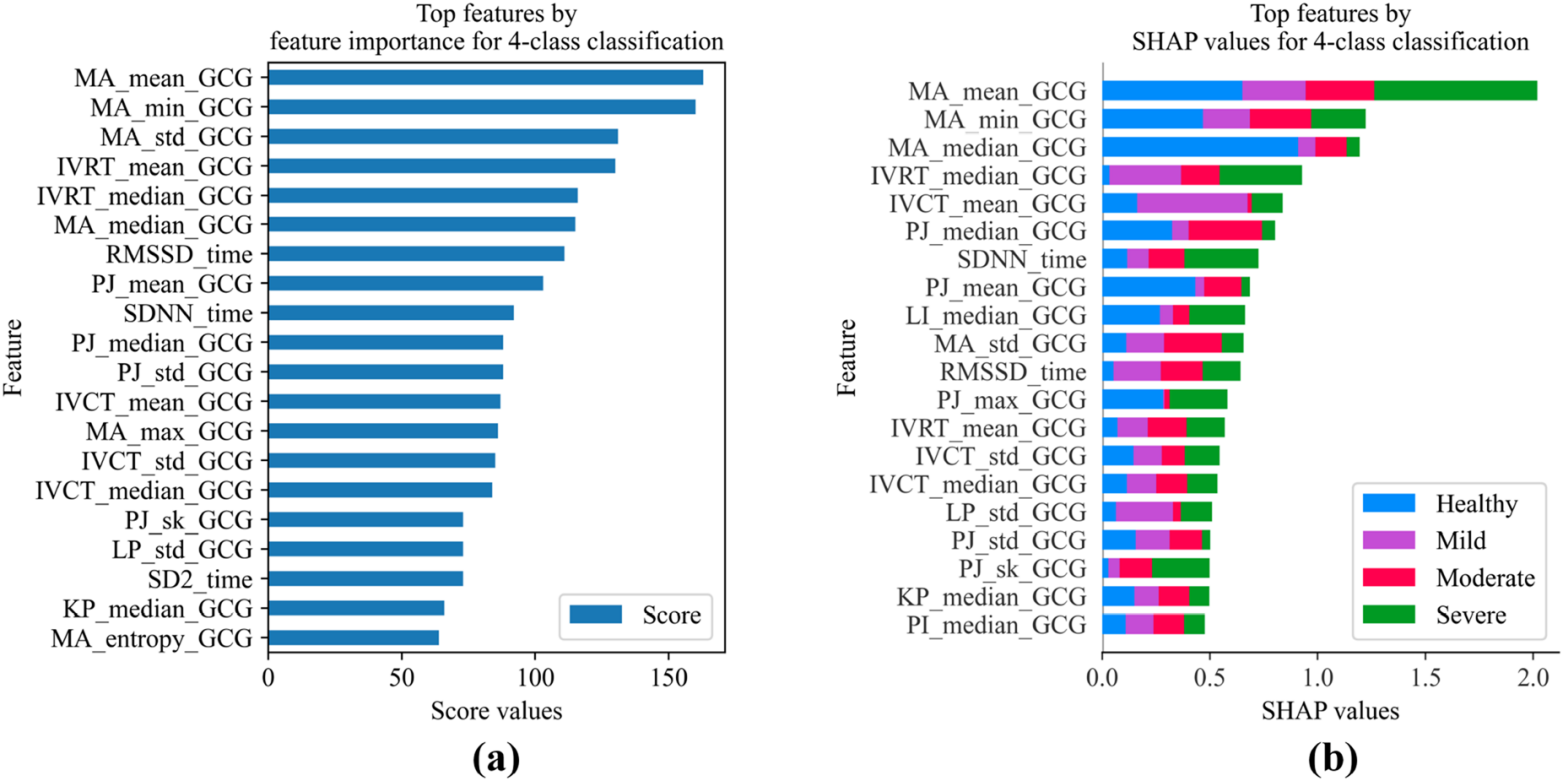
Top features for the classification of aortic stenosis severity level. (**a**) Feature importance by XGBoost, and (**b**) SHAP values.

Similar to AS detection task, the top features for the 4-class classification were obtained through the feature importance and the SHAP value methods, as summarized in Fig. 10a and b, respectively. By comparing the two feature sets, a high consistency can be observed in 16 common features, most of which are from the GCG timing intervals. Fig. 10b also contributes to interpreting the impact of each feature on the predicted classes. As shown, 5 MA-related features are among the top features, which demonstrates their importance in classifying severity level. According to Fig. 10b, MA_mean, IVRT_median, SDNN, and PJ_skewness indicate higher consistencies with severe cases than other features, which can be considered for future studies on AS. Certain features such as SDNN, MA_std, IVCT_std, and IVCT_median contribute to both healthy and mild classes equally, which may explain the 8.97% of the mild cases misclassified as healthy.

### Comparison with literature

AS detection is considered only in a limited number of research works, and this motivates the authors to compare the proposed framework with the works addressing heart disease detection/classification through SCG/GCG modalities. Therefore, the proposed method is compared with other methods in the literature for detecting AS^21,22^, as well as the works using SCG/GCG for the detection of other types of cardiovascular diseases (CVDs) including atrial fibrillation (AFib) and acute myocardial infarction^52,55–57^. The results are summarized in Table 7. The methods are compared in terms of their target CVDs, classifier, performance metrics, need for auxiliary signals other than SCG/GCG for feature extraction, and computational complexity. It should be noted that the proposed method is of low computational complexity due to using merely single-dimensional signal analyses, whereas some of literature methods which offer high performances tend to employ two-dimensional transformations, such as continuous wavelet transform (CWT)^21,22^. The results of our method at the subject level are superior to those of all previous works (F1-score: 1.00 vs. 0.88^55^, 0.95^22^, 0.96^56^, 0.96^57^, and 0.98^21^), despite the low computation power required by our method. It stands to reason that the lower the complexity, the more applicable the algorithm to real-time applications, which is a crucial point for wearable sensing systems. Even at the chunk level, our method performs equally or more accurately than other methods (F1-score: 0.96 vs. 0.88^55^, 0.95^22^, 0.96^56^, and 0.96^57^), except for the method proposed in^21^. However, our method predicts AS without any auxiliary signals, whereas in^21^, the ECG signal was used for feature extraction. Furthermore, the proposed framework suggests an analogous or higher F1-score, i.e., 0.96, and recall, i.e., 0.97, in comparison to the methods for detection of other CVDs. Furthermore, the proposed framework goes further beyond the disease detection by offering explainable modeling using SHAP values and feature importance from XGBoost to extract the physical meaning of the readings. To recapitulate, one could appreciate the proposed method as an accurate, computationally-efficient, and interpretable approach for AS detection which contributes to low-cost wearable sensing systems.

**Table 7:** Comparison of the model with previous works in the literature.

Signal type	Target CVD	Classifier	Accuracy	Precision	Recall	F1-score	Auxiliary	Computation	Reference
SCG + GCG	Acute myocardial	Kernel SVM	N/A	0.95	0.82	0.88	No	Low	^55^
SCG + GCG	AFib	Kernel SVM	0.98	N/A	0.97	N/A	No	Low	^52^
SCG + GCG	AFib	Kernel SVM	0.95	0.92	0.90	0.96	No	Low	^56^
SCG + GCG	AFib	Kernel SVM	0.97	1.00	0.93	0.96	No	Low	^57^
SCG + GCG	AS	RF	0.98	0.99	0.98	0.98	Yes (ECG)	High	^21^
SCG + GCG	AS	XGBoost	0.93	0.95	0.95	0.95	No	High	^22^
**SCG + GCG**	**AS**	**XGBoost**	**1.00**	**1.00**	**1.00**	**1.00**	**N** **o**	**Low**	**Proposed** **(subject)**
**SCG + GCG**	**AS**	**XGBoost**	**0.96**	**0.95**	**0.97**	**0.96**	**N** **o**	**Low**	**Proposed** **(chunk)**

Significant values are in bold.

## Conclusions and future work

This paper reports on the design and development of a novel reference-less framework for the detection of aortic stenosis based on SCG and GCG morphological characteristics and HRV parameters. The model is optimized in terms of filter design, and two groups of datasets are prepared at the subject and chunk levels. Furthermore, new parameters namely MA and P-included intervals are also introduced and shown to have higher consistencies with AS among the top features ranked by SHAP values and f-scores by XGBoost. Other features, such as time-domain and frequency-domain HRV parameters, are also extracted. However, a low correlation is demonstrated between HRV parameters and AS. On the contrary, ML models trained on the GCG timing intervals perfectly discriminate the AS cohort from the non-AS group. The most accurate ML model for both datasets is XGBoost, where F1-scores of 100.00% and 96.49% are reported for subject-level and chunk-level analyses, respectively. It is shown that the proposed optimized-filter design is suitable at both the subject-level and chunk-level settings, driving our methods to outperform previous works in the literature. Finally, the proposed framework was demonstrated to be robust enough for classifying the severity level of AS by offering 93.80% and 92.29% accuracy and F1-score, respectively.

In this work, data were collected from two groups of AS and non-AS subjects at senior ages. Future work involves a larger number of subjects by including subjects at younger ages. Also, due to the close relationship between AS and GCG parameters, there is promise in tracking the progress and severity of aortic stenosis at different stages using gyroscopic parameters.
